# A Novel Needle Mouse Model of Vascular Cognitive Impairment and Dementia

**DOI:** 10.1523/JNEUROSCI.0282-23.2023

**Published:** 2023-11-01

**Authors:** Zhongfang Weng, Catherine Cao, Nadezda A. Stepicheva, Fenghua Chen, Lesley M. Foley, Sarah Cao, Mohammad Iqbal H. Bhuiyan, Qingde Wang, Yuan Wang, T. Kevin Hitchens, Dandan Sun, Guodong Cao

**Affiliations:** ^1^Department of Neurology, University of Pittsburgh, Pittsburgh, Pennsylvania 15260; ^2^Geriatric Research Education and Clinical Center, Veterans Affairs Pittsburgh Healthcare System, Pittsburgh, Pennsylvania 15240; ^3^Animal Imaging Center, University of Pittsburgh, Pittsburgh, Pennsylvania 15203; ^4^School of Arts & Science, University of Washington in St Louis, St. Louis, Missouri 63130; ^5^Department of Pharmaceutical Sciences, The University of Texas at El Paso, El Paso, Texas 79902; ^6^Department of Surgery, University of Pittsburgh, Pittsburgh, Pennsylvania 15260; ^7^Department of Neurology, Xuanwu Hospital, Capital Medicine University, Beijing 100053, China; ^8^Department of Neurobiology, University of Pittsburgh, Pittsburgh, Pennsylvania 15213

**Keywords:** animal model, chronic cerebral hypoperfusion, cognitive stenosis, VCID, white matter injury

## Abstract

Bilateral common carotid artery (CCA) stenosis (BCAS) is a useful model to mimic vascular cognitive impairment and dementia (VCID). However, current BCAS models have the disadvantages of high cost and incompatibility with magnetic resonance imaging (MRI) scanning because of metal implantation. We have established a new low-cost VCID model that better mimics human VCID and is compatible with live-animal MRI. The right and the left CCAs were temporarily ligated to 32- and 34-gauge needles with three ligations, respectively. After needle removal, CCA blood flow, cerebral blood flow, white matter injury (WMI) and cognitive function were measured. In male mice, needle removal led to ∼49.8% and ∼28.2% blood flow recovery in the right and left CCA, respectively. This model caused persistent and long-term cerebral hypoperfusion in both hemispheres (more severe in the left hemisphere), and WMI and cognitive dysfunction in ∼90% of mice, which is more reliable compared with other models. Importantly, these pathologic changes and cognitive impairments lasted for up to 24 weeks after surgery. The survival rate over 24 weeks was 81.6%. Female mice showed similar cognitive dysfunction, but a higher survival rate (91.6%) and relatively milder white matter injury. A novel, low-cost VCID model compatible with live-animal MRI with long-term outcomes was established.

**SIGNIFICANCE STATEMENT** Bilateral common carotid artery (CCA) stenosis (BCAS) is an animal model mimicking carotid artery stenosis to study vascular cognitive impairment and dementia (VCID). However, current BCAS models have the disadvantages of high cost and incompatibility with magnetic resonance imaging (MRI) scanning due to metal implantation. We established a new asymmetric BCAS model by ligating the CCA to various needle gauges followed by an immediate needle removal. Needle removal led to moderate stenosis in the right CCA and severe stenosis in the left CCA. This needle model replicates the hallmarks of VCID well in ∼90% of mice, which is more reliable compared with other models, has ultra-low cost, and is compatible with MRI scanning in live animals. It will provide a new valuable tool and offer new insights for VCID research.

## Introduction

Vascular cognitive impairment and dementia (VCID) is the second leading cause of dementia after Alzheimer's disease (Duncombe et al., 2017) and is characterized by cerebral hypoperfusion, white matter injury (WMI), microinfarcts, reactive gliosis and cognitive dysfunction. Although the causes of VCID are unclear, increasing evidence suggests that chronic cerebral hypoperfusion (CCH) is the dominant pathogenic process that leads to WMI, which, in turn, strongly correlates with VCID (Longstreth et al., 1996; Tomimoto et al., 2003; The et al., 2011; Duncombe et al., 2017).

The bilateral common carotid artery (CCA) stenosis (BCAS) model closely replicates most characteristic features of VCID. Currently, there are two BCAS models, and both are expensive and incompatible with live magnetic resonance imaging (MRI) scanning because of metal implantation. The most commonly used microcoil model involves placing same-sized microcoils around both common carotid arteries (CCAs; Shibata et al., 2004), leading to symmetric stenosis on both sides. The drawbacks of the microcoil model are that the mortality rate is high if stenosis is severe, or there is a lack of long-term outcomes if stenosis is moderate. In contrast, the ameroid constrictor/microcoil model established by Hattori (Y. Hattori et al., 2015) causes asymmetric bilateral CCA stenosis (ABCS). In this model, an ameroid constrictor is implanted around the left CCA, leading to gradual occlusion on the left, while a microcoil is placed around the right CCA, leading to ∼50% blood flow reduction on the right. Because of occlusion of the left CCA, the mortality rate is ∼30–40% based on our experience.

While these models are well established, there is always speculation about which model comes the closest to mimicking human CCH. Indeed, most patients with intracranial arteriosclerotic stenosis suffer from permanent stenosis without occlusion (Gorelick et al., 2008). Moreover, among patients who suffered from severe carotid stenosis, 11.7% had bilateral stenosis and 87.3% had stenosis only on one side (Chang et al., 2022). Here, we introduce the new ABCS model with unilateral severe stenosis that mimics the clinical pathophysiology of patients suffering from severe carotid stenosis better than other models that cause either bilateral severe stenosis or unilateral occlusion.

The model is created by ligating mouse CCAs to various needle gauges (32-gauge needle for the right CCA and 34-gauge needle for the left CCA) using suture, followed by immediate needle removal leaving the suture in place (defined here as needle ABCS model). Needle removal led to moderate stenosis with ∼50.2% blood flow reduction in the right CCA and severe stenosis with ∼71.8% blood flow reduction in the left CCA. The needle ABCS model caused permanent cerebral hypoperfusion, WMI, microinfarcts, reactive gliosis and cognitive dysfunction. The new needle ABCS model mimics well the clinical pathophysiology of CCH caused by severe carotid stenosis, has extremely low cost and is compatible with live-animal MRI.

## Materials and Methods

All animal protocols were approved by the Institutional Animal Care and Use Committee at the University of Pittsburgh and performed in accordance with the NIH *Guide for the Care and Use of Laboratory Animals*. The manuscript adheres to the ARRIVE guidelines for reporting animal experiments. Male and female C57BL/6J mice (The Jackson Laboratory) aged 10–12 weeks (weighing 25–30 g) were used. All the surgical materials were autoclaved, and the surgical procedures were performed under sterile conditions.

### Needle ABCS model

Mice were anesthetized with 3.5% isoflurane through a face mask, then incubated and ventilated with 1% isoflurane in a mixture of 25% O_2_ and 74% N_2_O. Rectal temperature was continuously monitored and maintained at 37–37.5°C using a heating pad and a heating lamp throughout the surgery. Mice were placed in a Kopf stereotaxic frame. The fur was shaved, and the surgical sites were sterilized with Betadine Solution (10% iodine) and deiodized with 70% ethanol. A midline cervical incision was made, and the soft tissues including bilateral thyroid glands over the trachea were gently retracted with micro forceps. The skin was pulled aside at both sides from the incision. Either the left or the right CCA was exposed and carefully isolated from the vagus nerve. A precut fragment of a syringe needle (32 gauge for the right CCA and 34 gauge for the left CCA) was placed parallel with the CCA, and then the CCA was ligated to the needle using 7–0 silk suture until no blood flow was observed. The needle was then immediately removed to recover partial blood flow, leaving the suture ring around the CCA. Using the same procedure, second and third ligations were performed 1 and 2 mm proximal from the first ligation site. The skin was then sutured and treated with 1- to 2-mm-thick EMLA cream (Sigma). The mice were placed on an isothermal pad at 37°C and were continuously monitored for 2 h before being returned to the animal facility. Sham animals were subjected to the same procedure as needle mice except no ligation was performed on the CCAs.

### Blood flow and cerebral perfusion monitoring

CCA blood flow before ligation and immediately after needle removal was measured using the Perivascular Flow Module (Transonic Systems) with a 0.5 PSB flow probe. The probe was sterilized with 70% ethanol, then placed externally to either the left or right CCA to measure the flow. Cerebral perfusion before the surgery, immediately after needle removal, and at different timepoints after the surgery was measured by two-dimensional laser speckle (PeriCam system). The skull was exposed by a midline cervical incision and cleaned with phosphate buffered saline. Silicon oil was applied to retain moisture. A charge-coupled device camera with a laser speckle imager was placed above the head to image cerebral blood flow. The skin was then sutured and treated with 1- to 2-mm-thick EMLA cream. The mice were placed on an isothermal pad at 37°C and continuously monitored for 2 h before being returned to the animal facility.

### Immunohistochemical staining

After blocking with 5% bovine serum albumin in PBS/0.3% Triton X-100 for 1 h, free-floating coronal sections (25 μm) were incubated with primary antibodies at 4°C overnight followed by the appropriate secondary antibodies for 1 h at room temperature. The following primary antibodies were used: rabbit anti-Iba1 (1:1000, Wako Diagnostics), goat anti-MBP (1:500, Santa Cruz Biotechnology), rabbit anti-NF200 (1:500, Abcam), and rabbit anti-GFAP (1:500, Dako). Images were acquired via EVOS FL fluorescent microscope (Invitrogen).

### Luxol fast blue (LFB) staining and cresyl violet staining

LFB staining was performed as described (Jing et al., 2014). Brain sections were immersed in LFB 0.1% alcohol solution at 56°C overnight and washed with distilled water. Sections were then incubated in 0.05% lithium carbonate, dehydrated through graded alcohols, then stained with 0.5% cresyl violet for 5 min, differentiated with 70% ethanol and mounted with Permount.

### Hematoxylin and eosin (H&E) staining

Coronal brain slices were stained with H&E and the images were acquired using an EVOS microscope with a light filter.

### Diffusion tensor imaging (DTI) of *ex vivo* brains

Six weeks after surgery, brains were perfusion fixed with 4% paraformaldehyde and stored in PBS for MRI. *Ex vivo* MRI/DTI was preformed using a Bruker AV3HD 11.7 T/89 mm vertical bore scanner, equipped with a 20 mm quadrature radio frequency coil and Paravision 6.0.1 software (Bruker BioSpin), as previously described (Q. Liu et al., 2021). Magnetic resonance imaging (MRI) was performed at 500 MHz using a Bruker AV3HD 11.7 T/89 mm vertical bore small-animal MRI scanner, equipped with a 20-mm quadrature radio frequency coil and Paravision 6.0.1 software (Bruker BioSpin). T2-weighted images were acquired using a rapid acquisition with relaxation enhancement (RARE) sequence, with the following parameters: echo time/repetition time (TE/TR) = 20/4000 ms, averages = 8, 160 × 160 matrix, 25 slices with a 0.5-mm slice thickness, a RARE factor = 4, and a field of view of 16 × 16 mm. A DTI data set covering the entire brain was collected using a multislice spin echo sequence with five reference and 30 noncollinear diffusion-weighted images with the following parameters: TE/TR = 22/2800 ms, two averages, matrix size = 160 × 160, field of view = 16 × 16 mm, 25 axial slices, slice thickness = 0.5 mm, b-value = 3000 s/mm^2^, and Δ/δ = 11/5 ms. DTI data were analyzed with DSI Studio (http://dsi-studio.labsolver.org/). Regions of interest were drawn for the corpus callosum (CC), external capsule (EC), internal capsule (IC), striatum (St), hippocampus (Hippo), cingulum (Cg), and fimbria (Fi) for both the left and right hemispheres. Fractional anisotropy (FA) values, axonal diffusivity (AD), radial diffusivity (RD) values, and mean diffusivity (MD) values were quantitatively measured and analyzed using one-way ANOVA as described (Q. Liu et al., 2021).

### Behavioral tests

Mice were pretrained for 3–7 d before surgery. Sensorimotor deficits were assessed zero, one, two, three, four, five, and six weeks after surgery. Cognitive deficits were assessed four to six weeks after surgery. An extended long-term behavior test was performed 24 weeks after surgery.

Sensorimotor deficits were assessed by the following three tests. (1) The adhesive tape removal test was performed as described (Chen et al., 2019). Briefly, a piece of adhesive tape (3 × 4 mm) was attached to the distal-radial region on the wrist of each forelimb. The time to touch and remove the tape from the forelimb was recorded during five trials per day for each forepaw, with a minimal interval of 5 min between consecutive trials. Mice were trained twice daily for 3 d before surgery, and the mean times on the last day served as preoperative baselines. (2) The rotarod test was performed (Gan et al., 2012) by placing mice on a rotating drum (model 47650; Ugo Basile) with a speed accelerating from 5 to 40 rpm within 5 min. The time lapsed before the animal fell off the drum (latency to fall) was recorded and expressed as the mean duration of time on the rotarod. (3) For the foot fault test, mice were placed on an elevated grid surface [30 cm (L) × 20 cm (W) × 30 cm (H)] with a grid opening of 1.5 × 1.5-cm square and videotaped for 2 min. A foot fault was defined as a mouse misplacing its right forepaw or hindpaw and falling through the grid. The videotapes were analyzed by a blinded investigator to count the number of total foot faults made in a total of 50 steps and expressed as a percentage of total steps.

Cognitive deficit was assessed by the following three tests. (1) Retention of passive avoidance test is a sensitive measurement of impairments in learning and memory in rodents that do not exhibit long-term sensorimotor deficits (K. Hattori et al., 2000). In brief, mice were placed in a brightly lit box (free of foot shock) that was separated from a dark box (equipped with shock capacity) by a sliding door. The latency to enter the dark box was recorded. Mice were then allowed to access the dark box and given a pretraining of a 0.25-mA electric foot shock for 2 s. The latency to re-enter the dark box was quantified 42 d after the surgery. (2) A modified Morris water maze test was performed as described (Stetler et al., 2008, 2012). A square platform (11 × 11 cm) was submerged in a pool. The mouse was placed into the pool to locate the hidden platform. The time to find the platform (escape latency) was recorded for each trial as “spatial learning.” Three trials were performed daily on days 22–26 after needle surgery. For spatial memory testing at day 27, the platform was removed, and the time spent in the target quadrant was measured and expressed as a percentage of the total testing time of 60 s. (3) Novel object recognition (NOR) was performed 42 d after the needle surgery as described (Del Pino et al., 2017). Mice were placed in a box with two identical objects (A and B) and allowed 10 min of exploration. After a 1-h break, mice were then placed in the box in which object B was replaced with a new object C and allowed a further 5-min period of exploration. The time taken to explore the new and old objects was measured and expressed as the discrimination index using the formula (Time_new_ – Time_familiar_)/(Time_new_ + Time_familiar_), where Time_new_ and Time_familiar_ represent the time spent in exploring the new and familiar object, respectively.

### Statistical analysis

Statistical analysis was performed using GraphPad Prism software (version 9.3). Data with normal distributions were expressed as the mean ± SEM. Differences among multiple groups were analyzed using one-way or two-way ANOVA followed by a Bonferroni/Dunn *post hoc* correction. Comparisons between two experimental groups were analyzed using a two-tailed *t* test. A *p*-value of <0.05 was considered statistically significant.

## Results

### Optimization of needle gauges and ligation counts

**Figure 1.**
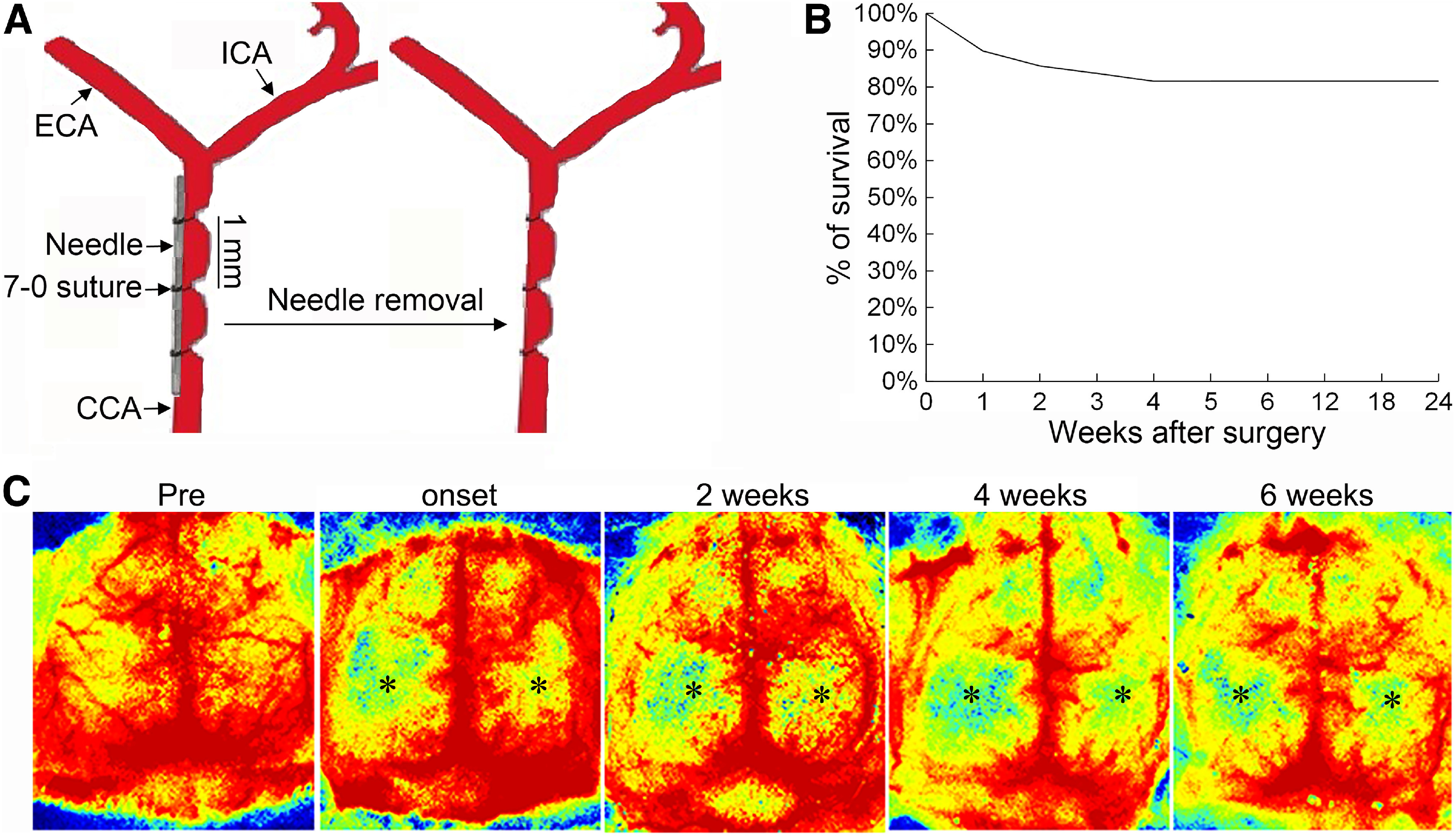
Persistent and long-term cerebral hypoperfusion in the needle ABCS model. ***A***, Schematic representation of the needle ABCS model. ***B***, Survival rate over 24 weeks after surgery. ***C***, Dynamic monitoring of brain blood flow with two-dimensional laser speckle presurgery, at onset, and two, four, and six weeks after surgery in the same animal, showing persistent and long-term cerebral hypoperfusion in both hemispheres, but being more severe in the left hemisphere. Black stars denote brain regions with hypoperfusion. CCA - common carotid artery, ECA - external carotid artery, ICA - internal carotid artery.

To better mimic human patient CCH, we established a new ABCS model by ligating left and right CCAs to different gauge needles to block blood flow (Fig. 1*A*). After confirming that the blood flow had been temporarily stopped by ligation, the needle was immediately removed, leaving the knotted suture band on the CCA, thus resulting in partial recovery of blood flow.

In clinic, 50–70% cranial stenosis is defined as moderate stenosis, while over 70% is referred to as severe stenosis. Therefore, we aimed at 50–80% blood flow reduction in the CCAs and thus needed to determine the best needle gauge capable of producing such an effect. Internal diameters (IDs) of mouse CCAs were measured with the Prospect T1 ultrasound system (S-Sharp Corporation). The average (*n* = 6) CCA ID was 0.41 ± 0.10 mm for 20–25 g mice, 0.47 ± 0.11 mm for 25–30 g mice, and 0.51 ± 0.08 mm for mice over 30 g. We mainly focused on mice weighing 25–30 g (10–12 weeks old). Based on the outer diameter (OD) of different needle gauges, we calculated the percentage of theoretical reduction of the lumen area after ligating the CCA to the needle (Table 1), and needles with gauges 30–34 were selected for the initial assessment.

**Table 1.** Effect of needle gauge and counts of ligations on blood flow reduction in the mouse left CCA

Needle gauge	Needle OD (mm)	% of CCA lumen area reduction by needle (lumen area-needle area)/lumen area x%	Blood flow reduction after 1st ligation	Blood flow reduction after 2nd ligation	Blood flow reduction after 3rd ligation
#30	0.31	48%	8.8%	22.8%	28.7%
#31	0.26	63.4%	13.4%	24%	29.8%
#32	0.23	71.4%	25.7%	43.8%	54.0%
#33	0.21	76.1%	40.3%	54.8%	68.2%
#34	0.19	80.5%	43.3%	61.5%	75.6%

We found that a single ligation only slightly reduced blood flow. A possible explanation is that a single stenosis might increase local pressure, leading to a compensatory increase in blood flow velocity. To reduce the contribution of local pressure on blood flow velocity, we therefore performed two additional ligations, 1 mm apart from each other. As shown in Table 1, each additional ligation further decreased CCA blood flow. Blood flow was reduced by 75.6% when the CCA was ligated three times to the 34-gauge needle, which is close to the predicted 80.5% reduction of lumen area. We therefore adopted three ligations (3L) for subsequent experiments. Further, we found that 3L of the left CCA with 34 gauge and 3L of the right CCA with 32 gauge caused persistent cerebral hypoperfusion and white matter damage, and we adopted this ligation combination (34-3L/32-3L) for the subsequent experiments.

### The needle ABCS model causes persistent and long-term cerebral hypoperfusion

We then determined blood flow, cerebral perfusion, and survival rate in the 34-3L/32-3L needle ABCS model. Blood flow was reduced by 71.8% and 50.2% in the left and right CCA, respectively (Table 2). Dynamic monitoring of brain blood flow with two-dimensional laser speckle showed that this model causes persistent cerebral hypoperfusion in both hemispheres, being more severe in the left hemisphere. Importantly, similar hypoperfusion was retained for over six weeks (Fig. 1*C*). The survival rate over 24 weeks was 81.6% (40 out of 49 survived; Fig. 1*B*) and death mainly occurred in the first week after surgery (55.5%).

**Table 2.** Average blood flow before and after ligation with 34-3L/32-3L in left and right CCA

	Left CCA	Right CCA
Preligation blood flow (ml/min)	0.955	0.989
Blood flow after ligations (ml/min)	0.27	0.493
% of blood flow reduction	71.8%	50.2%

### The needle ABCS model causes brain injury mainly in white matter regions

**Figure 2.**
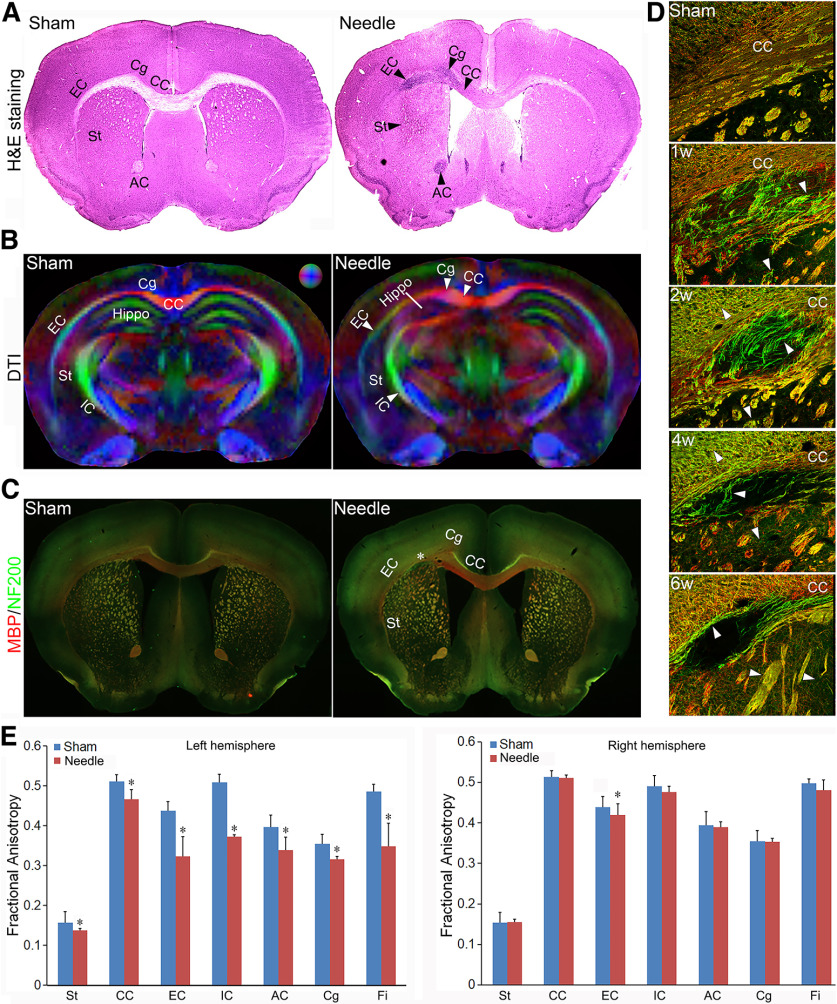
WMI in the needle ABCS mode. ***A***, Representative H&E staining of brain slices in sham and needle mice six weeks after surgery. Black arrowheads denote cell death in the CC, EC, IC, St, and AC in the left hemisphere in needle mice. ***B***, directionally encoded color (DEC) map of *ex vivo* DTI six weeks after surgery. The color sphere represents the directionality of the principal axis of diffusion (red = left/right, green = dorsal/ventral, and blue = rostral/caudal). White arrowheads denote anisotropy reduction in the left CC, Cg, EC, IC, St, and Hippo. ***C***, Representative MBP (red) and NF200 (green) staining in sham and needle mice six weeks after surgery (4×). Star represents microinfarct area. ***D***, Representative MBP (red) and NF200 (green) staining (200×) in left CC at different time points after needle surgery. White arrowheads denote axonal demyelination. ***E***, Quantitative analysis of FA in sham and needle mice. FA data in the CC, EC, IC, St, Hippo, Cg, and Fi for both the left and right hemispheres in sham and needle mice were quantitatively measured using DSI Studio and analyzed using one-way ANOVA. Data were expressed as mean ± SEM, *n* = 3 for sham, *n* = 5 for needle mice, * represents *p* < 0.05 versus sham.

H&E staining indicated that massive cell death occurred in CC, Cg, EC, St, AC, and Fi in needle mice (Fig. 2*A*), suggesting brain injury in these regions. Structural damage was obvious in the DTI images of the needle mice six weeks after the surgery when compared with the sham (Fig. 2*B*). Needle mice exhibited significantly lower FA in the EC, CC, IC, St, Hippo, Cg, and Fi in left hemisphere (Fig. 2*B*). Quantitative analyses showed significantly reduced FA in the EC, CC, IC, St, Hippo, Cg, and Fi in the left hemisphere (Fig. 2*E*). The needle mice also demonstrated a significant increase in radial diffusivity in the above regions, indicating demyelination (Alexander et al., 2007). A significant decrease in mean diffusivity was also seen in the above regions, which is inversely related to a loss of cells (Stolp et al., 2018). A decrease in axonal diffusivity was seen in the above regions, suggesting axonal injury. However, only EC showed FA changes in the right hemisphere (Fig. 2*E*), suggesting that the needle ABCS model causes brain injury mainly in white matter regions in the left hemisphere. We further analyzed demyelination with double labeling of MBP and NF200, markers of myelin and axons, respectively. As shown in Figure 2*C*, more green stained axons and fiber bundles were found in CC, EC, and St, suggesting axonal demyelination. The integrity of fiber bundles in the striatum was also compromised in needle mice (Fig. 2*D*). Microinfarct, the hallmark of VCID, was found in the left EC region, as denoted by increased cell death and loss of MBP (myelin) and NF200 (axons) staining (Fig. 2*C*,*D*). Single or multiple microinfarcts were also detected in other regions including St and EC (data not shown).

### The needle ABCS model causes neuroinflammation and astrocyte activation

**Figure 3.**
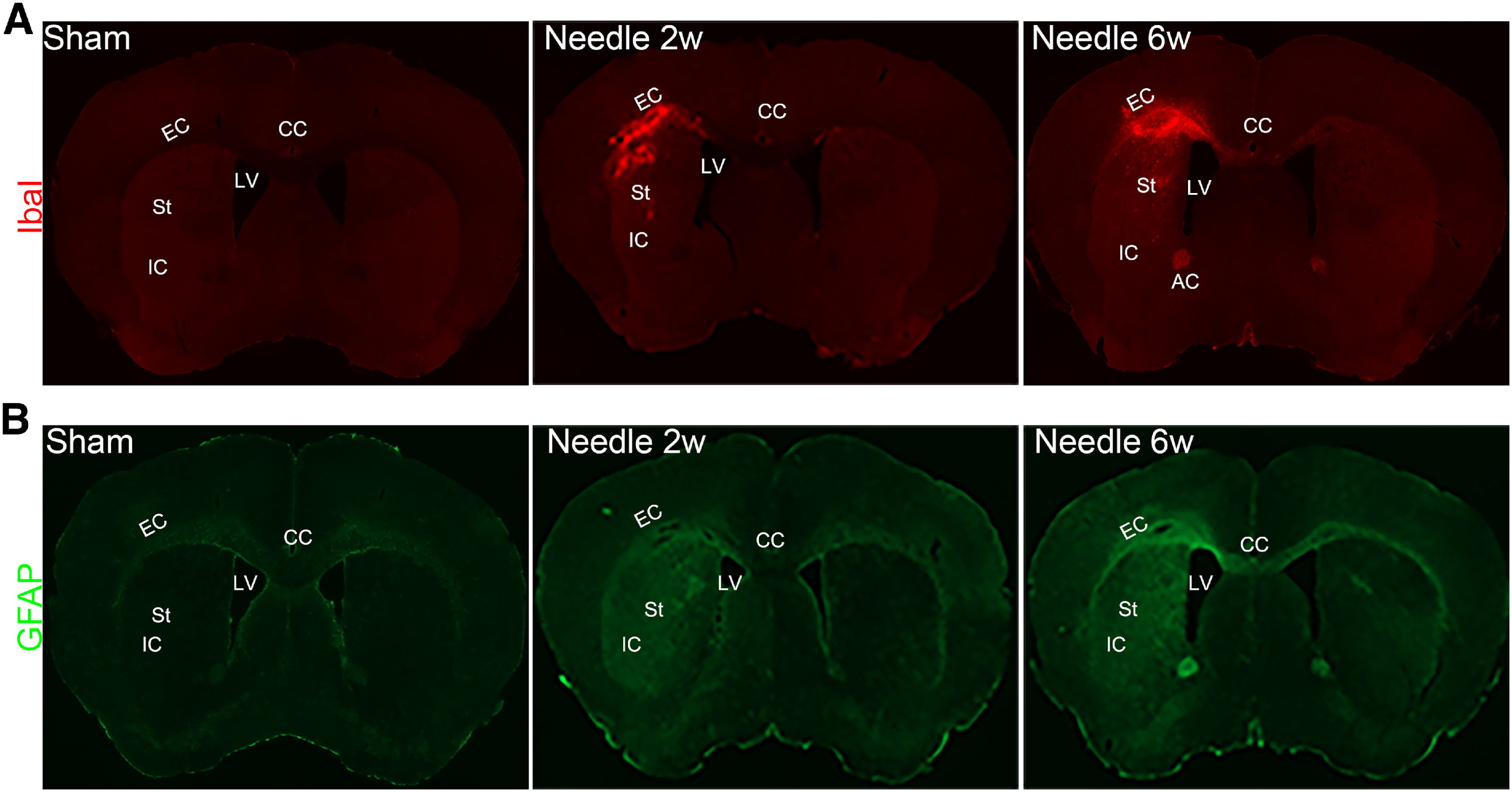
Microglia and astrocyte activation in the needle ABCS model. ***A***, Representative IbaI staining two and six weeks after surgery, showing massive microglia activation in left CC, EC, St, and IC in needle mice. LV, left ventricle. ***B***, Representative GFAP staining two and six weeks after surgery, showing astrocyte activation in left CC, EC, St, and IC in needle mice.

Microglia activation is a sensitive marker for brain inflammation and injury. As shown in Figure 3*A*, the needle ABCS model caused massive activation of microglia/macrophages in the EC, CC, IC, and St in the left hemisphere, as supported by IbaI staining, suggestive of neuroinflammation and potential brain injury in these regions. Moreover, massive astrocyte activation (indicated by increased GFAP staining) was observed in the CC, EC, IC, and St in the left hemisphere in needle mice (Fig. 3*B*). Meanwhile, only weak GFAP expression was detected exclusively in the CC region in sham mice (Fig. 3*B*).

### The needle ABCS model causes sensorimotor and cognitive impairment

**Figure 4.**
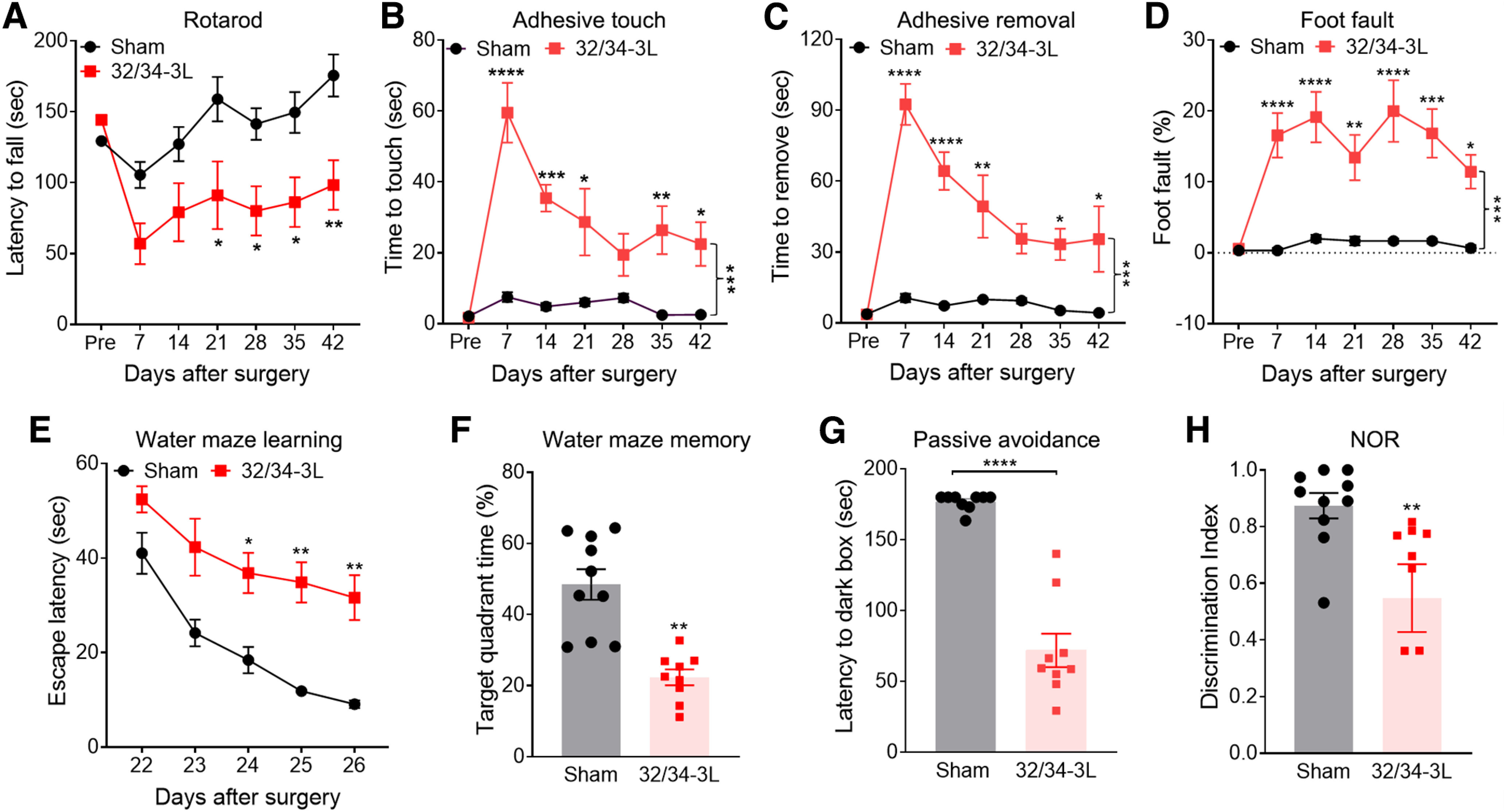
Sensorimotor and cognitive impairment in the needle ABCS model. ***A***, Rotarod test from onset to six weeks. ***B***, ***C***, Adhesive tape touch and removal test. ***D***, Foot-fault test. ***E***, ***F***, Spatial learning and memory in water maze test at fourth week. ***G***, Passive avoidance test at sixth week. Mice in the dark box were given a pretraining with a 0.25-mA electric foot shock for 2 s. On day 7, the latency to access the dark box was measured. ***H***, NOR test at sixth week. Data were expressed as mean ± SEM, *p < 0.05 needle versus sham; **p < 0.01 versus sham; ***p < 0.001 versus sham; ****p < 0.0001 versus sham, two-way ANOVA and Neuman–Keuls *post hoc*. *n* = 10 for sham, *n* = 10 for needle groups.

The needle ABCS model mainly damages WM regions, which are strongly associated with cognition. Therefore, we assessed both sensorimotor and cognitive functions. As shown in the rotarod test in Figure 4*A*, needle mice showed decreased latency to fall from the rod as compared with sham mice starting the third week after the surgery, suggesting motor dysfunction. In the adhesive tape removal test (Fig. 4*B*,*C*), needle mice spent a significantly longer time to sense and to remove the tape starting the first week after the surgery as compared with sham. Similarly, needle mice made more foot faults misplacing the right paw and fell through the grid more often (Fig. 4*D*). These data suggest that needle surgery causes lasting sensorimotor dysfunction.

In the water maze test, needle mice spent a longer time locating the submerged platform (Fig. 4*E*) and less time in the goal quadrant (Fig. 4*F*) compared with sham mice four weeks after surgery, suggesting impaired spatial learning and memory. In the passive avoidance test, the latency to enter the dark box was 184 ± 18 versus 81 ± 11 s for sham versus needle mice, respectively, suggesting that sham mice learned that the dark box was associated with the foot shock and therefore passively avoided that area. In contrast, needle mice displayed learning/memory impairments and accessed the dark box more frequently despite being shocked there (Fig. 4*G*). In the NOR test, sham animals exhibited a preference to explore the new object, while needle mice lost interest in exploring the new object (Fig. 4*H*), further confirming cognitive deficits.

### The needle ABCS model causes long-term cerebral hypoperfusion, WMI, and cognitive impairment

**Figure 5.**
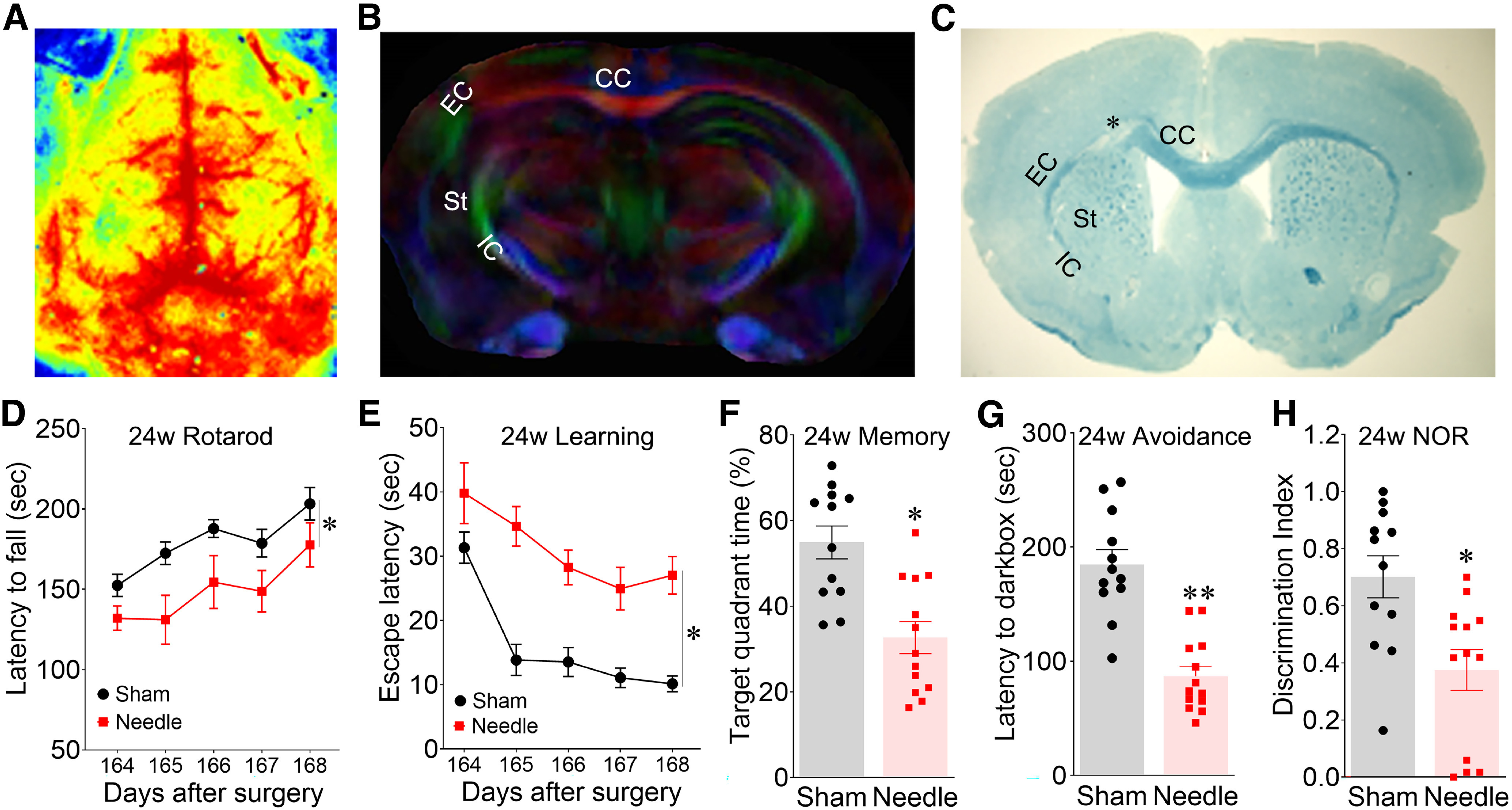
Persistent and long-term cerebral hypoperfusion, WMI and cognitive impairment at 24 weeks in the needle ABCS model. ***A***, Two-dimensional laser speckle image of brain perfusion in needle mice 24 weeks after surgery. ***B***, DEC map of *ex vivo* DTI in needle mice 24 weeks after surgery. ***C***, LFB staining. * denotes microinfarct. ***D***, Rotarod test. ***E***, ***F***, Spatial learning and memory in water maze test 24 weeks after the surgery. ***G***, Passive avoidance test. ***H***, NOR test. Data were expressed as mean ± SEM, *p < 0.05 needle versus sham; **p < 0.01 versus sham, two-way ANOVA and Neuman–Keuls *post hoc*. *n* = 12 for sham, *n* = 13 for needle groups.

Another drawback of the classical BCAS model is the potential disappearance of long-term outcomes; therefore, we further investigated the long-term outcomes of the needle technique 24 weeks after surgery. Cerebral hypoperfusion (Fig. 5*A*), WMI (Fig. 5*B*,*C*), sensorimotor (Fig. 5*D*), and cognitive impairment (Fig. 5*E–K*) were still clearly detected 24 weeks postsurgery.

### The needle ABCS model causes relatively milder brain injury in female mice compared with males

**Figure 6.**
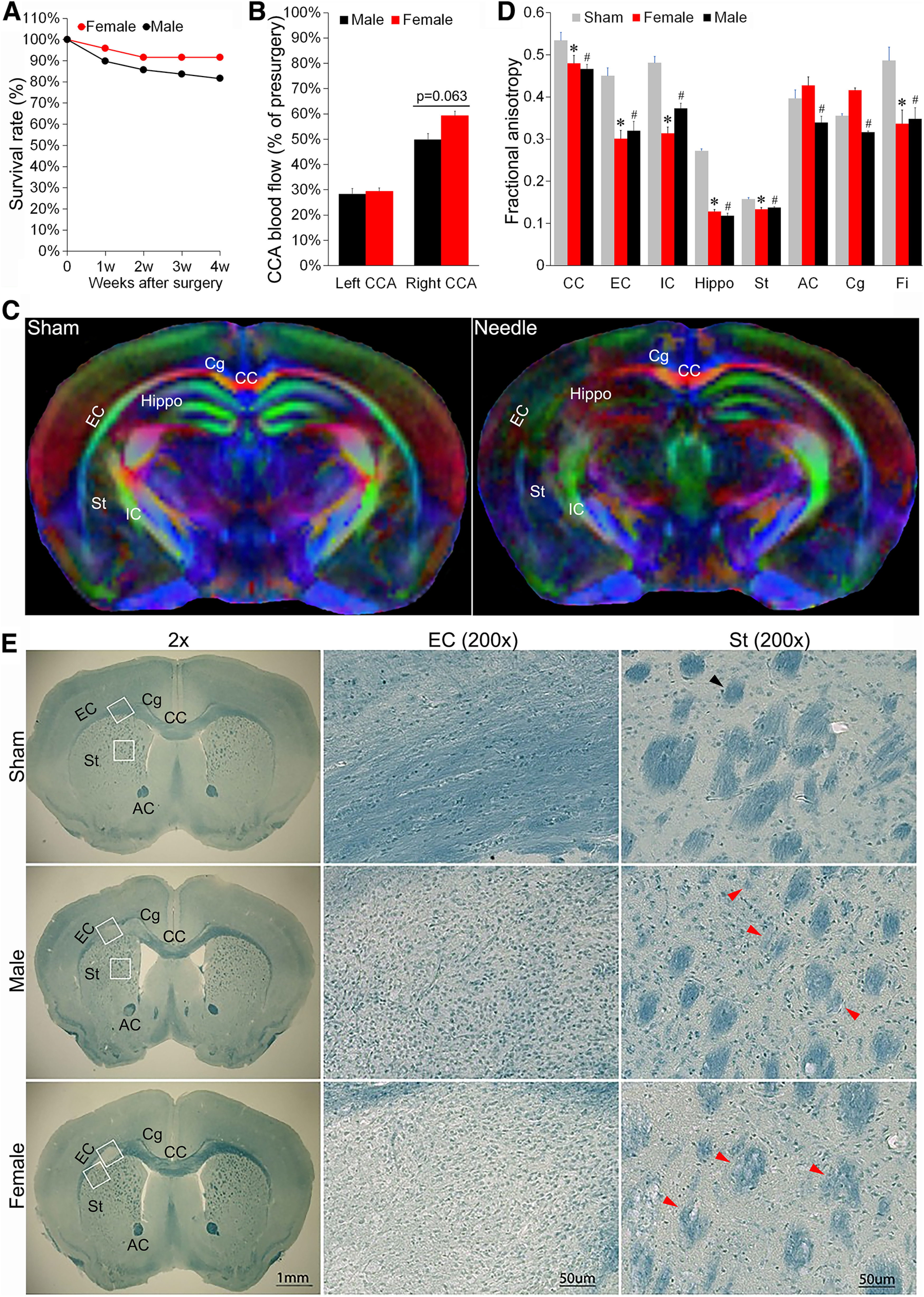
The needle ABCS model causes relatively milder brain injury in female mice than in male. ***A***, Survival rate over four weeks after surgery in female and male mice. ***B***, CCA blood flow after surgery in male and female mice. ***C***, DEC map of *ex vivo* DTI in female mice four weeks after surgery. ***D***, Quantitative analysis of FA in sham, female and male needle mice. *n* = 4 for sham (mixed gender), *n* = 4 for female. *n* = 4 for male. *p < 0.05 female versus sham. #*p* < 0.05 male versus sham. ***E***, Representative LFB staining of brain slices in sham, female and male needle mice four weeks after the surgery. White box in the left panel denotes the location of a higher definition image (200×) from EC (middle panel) and St (right panel). Black arrowhead denotes normal fiber bundle, red arrowheads denote disintegrated and demyelinated fiber bundle.

Recent studies indicate that gender is a critical factor in ischemic stroke, and that women after menopause have increased stroke incidence and more severe outcomes as compared with men (Roquer et al., 2003; Andersen et al., 2005). Thus, we determined whether gender influences blood flow, brain injury and cognitive function in the needle model. The four-week survival rate (Fig. 6*A*) for female mice subjected to needle ABCS surgery was 91.6% (22 out of 24), which is higher than that of male mice (81.6%, 40 out of 49). Left CCA blood flow (Fig. 6*B*) was reduced to 29.41% of the presurgery level after needle removal, similar to that of male mice (28.20%). Surprisingly, we found that right CCA blood flow in females was reduced to 59.29% of presurgery after needle removal, which was higher compared with male mice (49.8%), although it did not reach statistical significance (*p* = 0.062). Relatively higher right CCA blood flow after needle removal could partially explain the lower mortality of female mice compared with males. DTI images showed a similar white matter injury pattern in FA and radial, and mean and axonal diffusivity in the CC, EC, IC, Hippo, and Fi in the left hemisphere (Fig. 6*C*). Quantitative analysis showed that FA was significantly different in CC, EC, IC, St, Hippo, and Fi in both male and female needle mice as compared with sham. However, there are significant difference in FA in AC and Cg in male but not female mice (Fig. 6*D*). fast blue specifically stains myelin, and its intensity is proportional to myelin expression. As shown in Figure 6*E*, less and lighter blue staining in Cg, EC, IC, St, and AC in the left hemisphere was observed as compared with sham or corresponding regions in the right hemisphere, suggesting demyelination in these regions. The integrity of fiber bundle was compromised in St in both male and female needle mice. WMI was observed in ∼82% of female mice, which is slightly lower than that of 90% in male mice. NOR and passive avoidance tests showed similar cognitive deficits in female mice at four weeks after surgery (data not shown).

## Discussion

In this study, we mimicked CCH induced by arteriosclerotic stenosis and established a new asymmetric BCAS model by ligating left and right CCAs to 34- and 32-gauge needles, respectively. This technique caused persistent and long-term cerebral hypoperfusion in both hemispheres (being more severe in the left hemisphere), mainly damaged WM regions, and impaired cognitive function. This model replicates several key features of VCID including cerebral hypoperfusion, WMI, microinfarct, astrocyte activation and cognitive impairment. Importantly, cerebral hypoperfusion, WMI and cognitive impairment presented persistently in ∼90% of mice and lasted for up to 24 weeks. Thus, the needle ABCS model is a reliable model for studying both WMI and VCID.

The needle ABCS model has the following advantages over other BCAS models. (1) The needle ABCS model closely mimics the clinical pathophysiology of patients suffering from severe carotid stenosis. This conclusion is based on the reports that most patients suffering from arteriosclerotic stenosis present with permanent stenosis without occlusion (Gorelick et al., 2008) and that 87.3% of severe patients have unilateral stenosis (Chang et al., 2022). In contrast, the currently established methods cause either bilateral severe stenosis or unilateral occlusion. (2) The needle ABCS model replicates WMI and cognitive impairments more persistently in ∼90% of mice. A potential reason is that using three ligations leads to a fragment of stenosis rather than point stenosis. Moreover, if one ligation was loose, the other ligations could still secure the stenosis, thus increasing the reliability of the method. (3) Extremely low cost. Microcoil or ameroid restrictors are expensive and each BCAS mouse costs around $100, while the needle ABCS model uses only low-cost silk sutures (about $1). (4) The needle ABCS model causes asymmetric injury in the two hemispheres, allowing for special analytic approaches that relate to cerebral-spinal tract damage, such as the corner test, foot fault and cerebral-spinal tract tracing. (5) The gauge of needles could be adjusted based on the body weight of mice and/or specific requirements for the levels of blood flow reduction. (6) No metal is implanted, allowing for MRI monitoring of brain injury in live animals.

In our study, we found that a single ligation only slightly reduced the blood flow and that three ligations were required to reduce the blood flow to maintain persistent and long-term cerebral hypoperfusion (Table 1). As stated before, this can be potentially explained by the fact that a single ligation could cause a local increase in pressure at the ligation point, which results in an increase in blood flow velocity, thus compensating for the reduction of the blood flow. However, three ligations 1 mm apart lead to a fragment of stenosis, rather than to a point stenosis, thus increasing vascular resistance and reducing the local blood flow velocity. On the other hand, vascular tension restoration after needle removal may potentially loosen the suture tie. With three ligations instead of just one, loosening of one tie would not be as noticeable because of compensation from the remaining ties. A fragment of stenosis caused by 3L significantly increases the consistency of CCA stenosis among animals, leading to persistent cerebral hypoperfusion in ∼90% of animals. In clinic, CCA stenosis caused by atherosclerosis usually involves a fragment of CCA, not focal point stenosis. Thus, our needle ABCS model closely mimics CCA stenosis induced by atherosclerosis.

A syringe needle was also used to produce a rat BCAS model in Wang's study (Wang et al., 2020). The significant difference between Wang's needle model and our needle ABCS model is that equal gauge needles were used for both CCAs in Wang's needle model, leading to symmetrical hypoperfusion. Based on our experience, a significant disadvantage of equal hypoperfusion in bilateral CCA is that mortality is high if stenosis is severe, or pathologic/neurologic outcomes (especially long-term) are not sufficient if stenosis is moderate. In Wang's study, cognitive function was assessed between days 2 and 6 after the surgery, and mortality rate was assessed 30 d after the surgery, so long-term effects of their model still need further investigation. In our needle ABCS model, different gauge needles were used for the two CCAs, leading to moderate hypoperfusion in the right hemisphere and severe hypoperfusion in the left hemisphere. Animals survived better because of the moderate hypoperfusion in the right hemisphere, and the 24-week mortality rate was ∼18%. Persistent severe hypoperfusion in the left hemisphere produced long-term pathologic and neurologic outcomes up to 24 weeks. Meanwhile, asymmetric brain injury allowed for analyzing pathologic and behavioral outcomes with special analysis approaches.

One more important factor that makes the needle ABCS model stable (in addition to a fragment of stenosis) is contralateral CCA blood flow control. In our needle ABCS model, we found that right CCA blood flow control is critical, and that 50–60% blood flow reduction is required to cause severe hypoperfusion and brain damage in the left hemisphere. A potential reason is the establishment of collateral circulations after CCA stenosis. In line with this, in the bilateral occlusion mouse model (Tamaki et al., 2006), anastomosis of the pterygopalatine artery and the external carotid artery (ECA) was observed, bridging the internal carotid artery (ICA) and the ECA. The posterior communicating (Pcom) artery was absent in sham mice but appeared in bilateral occlusion mice, which enabled blood flow to the anterior circulation from the vertebrobasilar arteries. The bilateral occlusion mice that had visualized Pcom did not have any infarction. In human CCA occlusion (Wang et al., 2019), antegrade blood flow in ipsilateral ICA was established by retrograde perfusion of ECA through carotid bifurcation, and four collateral circulation pathways were observed. Because of these effective collateral circulations between left and right CCA, contralateral CCA blood flood control is critical to obtain ipsilateral cerebral hypoperfusion and brain damage.

Recent studies indicate that gender is a critical factor for ischemic stroke. Female mice are resistant to stroke at a young age, but become vulnerable to stroke with age (F. Liu et al., 2009). In line with this, we observed a higher survival rate in young female mice compared with male mice at four weeks after needle surgery and a slightly lower level of WMI in ∼82% of female mice as compared with 90% of male mice (Fig. 6). Female mice had similar blood flow in the left CCA, but slightly more blood flow in the right CCA compared with male mice after needle removal. We measured the ID of CCA in male mice, but we did not measure the ID of CCA in female mice. It is likely that female mice have relatively smaller ID of CCA than male mice, so removal of the same gauge needle could cause relatively less stenosis, leading to a relatively higher rate of blood flow in the right CCA. Further ID measurement in female mice may answer this question. Stroke pattern change over the lifespan in females is estrogen dependent (F. Liu et al., 2009), so likely less severity of WMI in females may also be related to estrogen. Utilization of ovariectomized female mice may provide evidence of whether a lower rate of white matter injury in female mice is caused by relatively higher CCA blood flow or estrogen. One interesting phenomenon we observed is that some female mice did not have visible brain tissue injury, but still showed cognitive impairment, suggesting that CCH may cause some undetectable injury that still affects cognitive networks/function. Accordingly, we observed similar cognitive deficits in male and female mice at four weeks after surgery. However, whether female mice maintain same long-term outcomes at an extended timeframe remains for further investigation.

It is widely accepted that CCA stenosis causes CCH and cerebral WM disease (Kandiah et al., 2014; Baradaran et al., 2016). Consistent with this, we found that brain injury induced by the needle ABCS model mainly occurred in the EC, CC, IC, and St in the left hemisphere, and was characterized by axonal demyelination and damage, disintegration of fiber bundle (Fig. 2), and reactive gliosis (Fig. 3). WM is involved in the relay of motor and sensory information to and from the cerebral cortex and determines cognitive behavior. Thus, WMI may disrupt motor and sensory function and cause neurobehavioral syndromes and cognitive impairment (Leys et al., 1999; Pantoni et al., 1999; Desmond, 2002). In line with this, we found that the needle ABCS model causes long-term sensorimotor and cognitive impairment (Fig. 4). Interestingly, hippocampal injury occurred in ∼20% of mice, and injury in cortex (data not shown) occurred in ∼10% of mice. The hippocampus is related to memory, and its injury likely contributes to the cognitive impairment observed in the needle ABCS model. However, mice with hippocampal or cortex injury usually showed severe and early neurologic dysfunction in neurobehavioral tests and could be easily distinguished from the mice with WMI only. We could further increase the group number and perform a parallel comparison between animals with WMI only and animals with combined WM and hippocampal injury, and this may provide new insights into WMI and cognitive dysfunction. In conclusion, a novel, low-cost, reliable VCID model compatible with live MRI scanning with long-term outcomes was established, and this may provide new insights for VCID investigation.
